# A passage-free, simplified, and scalable novel method for iPSC generation in three-dimensional culture

**DOI:** 10.1016/j.reth.2024.02.005

**Published:** 2024-03-10

**Authors:** Masaya Tsukamoto, Tomoyuki Kawasaki, Mohan C. Vemuri, Akihiro Umezawa, Hidenori Akutsu

**Affiliations:** a Center for Regenerative Medicine, National Center for Child Health and Development, 2-10-1 Okura, Setagaya, Tokyo 157-8535, Japan; b Thermo Fisher Scientific, 7335 Executive Way, Frederick, MD 21702, USA

**Keywords:** iPS cells generation, Reprogramming, 3D culture, Cell culture, Differentiation, Bioreactor

## Abstract

Induced pluripotent stem cells (iPSCs) have immense potential for use in disease modeling, etiological studies, and drug discovery. However, the current workflow for iPSC generation and maintenance poses challenges particularly during the establishment phase when specialized skills are required. Although three-dimensional culture systems offer scalability for maintaining established iPSCs, the enzymatic dissociation step is complex and time-consuming. In this study, a novel approach was developed to address these challenges by enabling iPSC generation, maintenance, and differentiation without the need for two-dimensional culture or enzymatic dissociation. This streamlined method offers a more convenient workflow, reduces variability and labor for technicians, and opens up avenues for advancements in iPSC research and broader applications.

## Highlights

• Successful iPSC generation in a physiologically relevant 3D culture system.
• Maintenance without single-cell dissociation; simplified workflow and minimal labor.
• Efficient differentiation of iPSCs: enhances disease modeling and drug discovery.
• Streamlined workflow: better efficiency and lower variability in iPSC research.

**eTOC blurb**

The current iPSC workflow is complex, time consuming, and prone to variability. This study introduces a new approach that eliminates the need for two-dimensional culture or enzymatic dissociation and simplifies iPSC generation, maintenance, and differentiation. Our streamlined method is convenient and paves the way for advancements in iPSC research and broader applications.

## 1 Introduction

Induced pluripotent stem cells (iPSCs) are generated from somatic cells and possess characteristics similar to embryonic stem cells (ESCs), making them useful for disease modeling, etiological studies, and drug discovery [18]. However, the workflow for iPSC generation presents challenges, particularly during the establishment phase when specialized skills are required [8,13]. Manual expertise is necessary to select primary iPSC colonies with a good morphology, and spontaneous differentiation is a common issue within the first few passages. These intricacies contribute to inherent variability, emphasizing the need for an improved iPSC workflow.

Maintaining iPSC lines under three-dimensional (3D) conditions offers scalability, which has been achieved using agitation rotor machines and bioreactors [5,10]. However, the enzymatic dissociation step for single-cell cultures under 3D conditions is time-consuming and complex ([[7], [10]]. Establishing a stable human iPSC generation system within 3D culture while preserving pluripotency remains challenging.

To address these issues and reduce technician variability and workload, a more convenient and streamlined iPSC workflow is necessary. This study aimed to develop a technique that enables iPSC generation, maintenance, and differentiation under 3D culture conditions, eliminating the need for two-dimensional (2D) culture and enzymatic cell dissociation. By simplifying this procedure, this method aims to enhance the utility of iPSCs in downstream applications.

## 2 Results

### 2.1 Adipose-derived mesenchymal stem cells reprogrammed to pluripotent state in the absence of 2D culture conditions

**Fig. 1:**
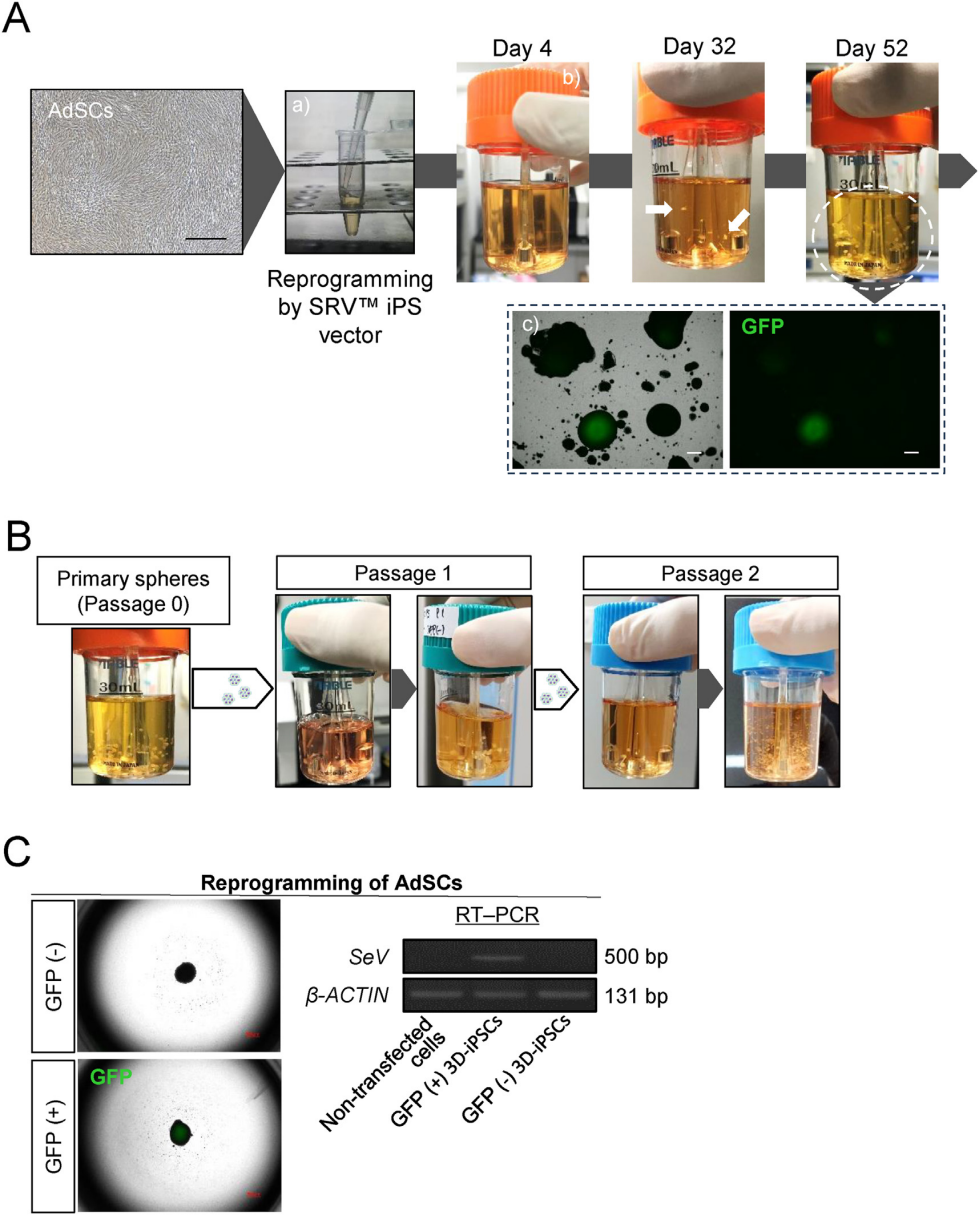
Reprogramming of somatic cells into iPSCs under 3D conditions (A) Representative images depicting the progression of reprogramming under three-dimensional (3D) conditions. Generation of induced pluripotent stem cells (iPSCs) by Sendai virus vectors (SRV™ iPSC Vector). Adipose-derived mesenchymal stem cells (AdSCs) were collected and suspended in a microtube with reprogramming factors for 2 h (a). Cells were then washed and cultured in a single-use bioreactor (b). White arrows indicate primary spheres. Cells were observed by fluorescence microscopy and green fluorescent protein (GFP) expression (Sendai virus vector remnant cells) noted. The sphere cells demonstrated substantial growth (c; white dashed circle) but certain clustered cells exhibited residual expression of GFP (black dashed rectangle). Black/white scale bars = 500 μm. (B) Growth appearance of 3D-iPSCs. Only a few spheres were transferred to the next bioreactor (white arrows). The cells increased in size and number during each passage (dark gray arrows). (C) Left images illustrate GFP-positive or -negative spheres. GFP-negative spheres indicate the absence of Sendai virus (*SeV*), as shown by quantitative reverse transcription polymerase chain reaction (RT–PCR) image (right panel). AdSCs were donor cells for iPSC reprogramming. *β-ACTIN* was the housekeeping gene.

To examine the feasibility of reprogramming human somatic cells under 3D culture conditions we performed experiments using human adipose-derived mesenchymal stem cells (AdSCs). Initially, we obtained an AdSC suspension from 2D-cultured cells through trypsinization. Subsequently, we introduced pluripotency-associated genes into detached AdSCs using Sendai virus vectors (SRV™ iPSC Vector, TOKIWA-Bio Inc., Japan) in suspension conditions for 2 h at 37 °C. Vector-transfected cells were then rinsed with phosphate-buffered saline (PBS), and cultured in 30-mL spinner flasks using a stirred bioreactor system [4]. Suspension cultures were grown in StemScale media [11]. To expedite the reprogramming process, we supplemented the medium with two small molecules, a Notch signaling inhibitor (N-[N-(3,5-difluorophenacetyl)-L-alanyl]-S-phenylglycine *t*-butyl ester [DAPT]) and a histone methyltransferase inhibitor (histone H3 methyltransferase disruptor of telomeric silencing 1-like inhibitor [iDOT1L]), based on our previous work [12]. Following approximately 30 days in culture, spheroid formations were observed (Fig. 1A). The number of spheres multiplied without cell dissociation procedures. Approximately 50 days after seeding, the cells reached confluence in the same reactor. Since SRV™ iPSC Vector contains Green Fluorescent Protein (GFP), SRV vector-positive cells could be positively identified with a GFP light source without the need for immunostaining. A spheroid with a GFP signal indicates the existence of Sendai virus vector remnant cells (Fig. 1A). The primary spheres grew in size and number without requiring enzymatic dissociation; therefore, we simply transferred a few spheroids to the next bioreactor as part of the passage procedure. The spheres maintained their growth throughout passage (Fig. 1B). Despite the extended culture period, we observed a mixture of GFP-negative and GFP-positive cells (Fig. 1C). Silencing exogenous genes is one of the criteria for complete cell reprogramming; therefore, we selectively passaged GFP-negative spheres. Upon subsequent cell growth, we confirmed the absence of Sendai virus (SeV) in the GFP-negative spheres (Fig. 1C).

We successfully demonstrated the reprogramming of somatic cells under 3D conditions. Reprogrammed cells can be propagated without the need for single-cell dissociation steps; instead, they can be transferred as spheres to the next bioreactor. Despite GFP-negative selection, our methodology effectively eliminated the complexity associated with iPSC generation and streamlined the cultivation process, thereby making it easily reproducible.

### 2.2 iPSCs generated under 3D conditions exhibit a pluripotent state

**Fig. 2:**
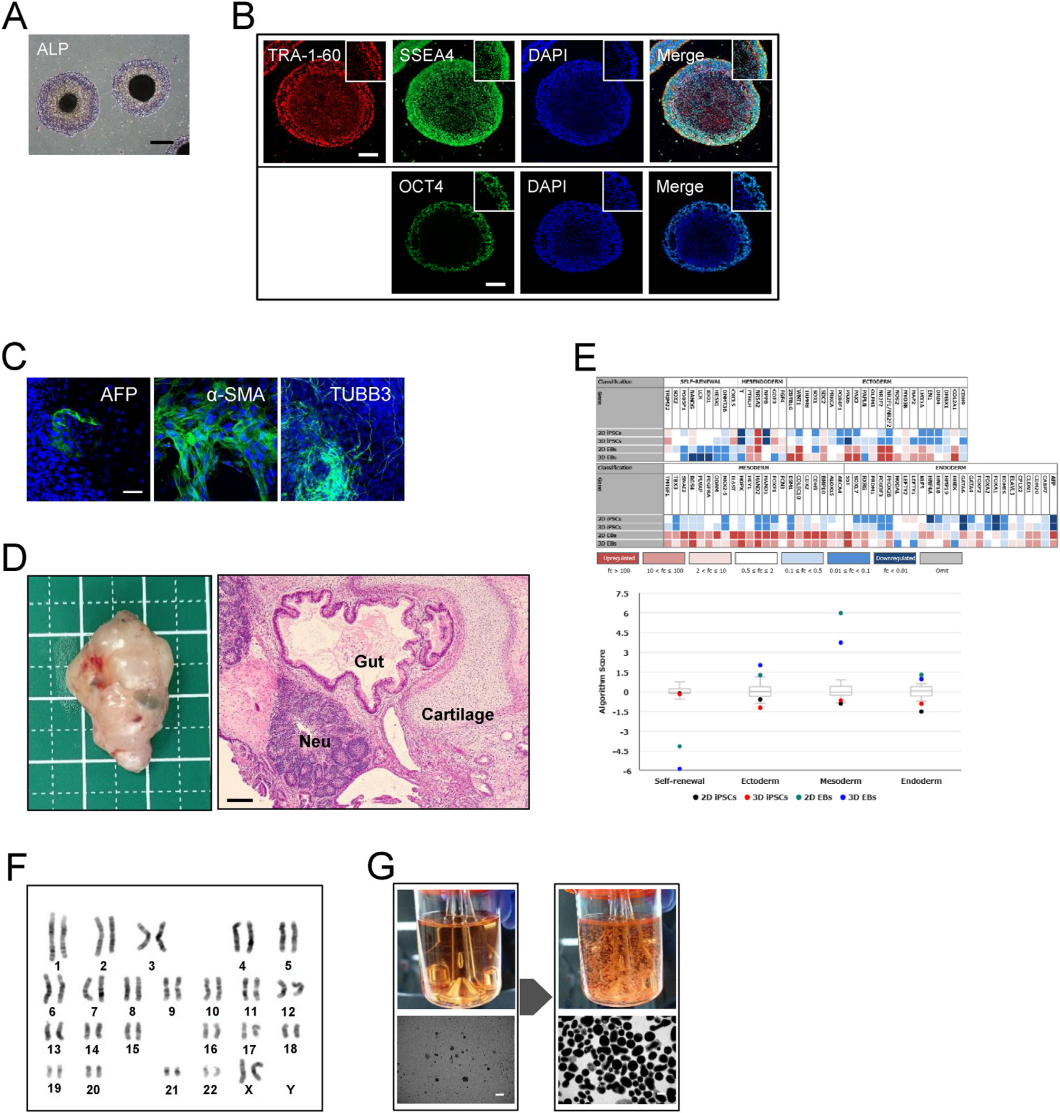
Characteristics of 3D-iPSCs (A) Alkaline phosphatase (ALP) staining of induced pluripotent stem cells (iPSCs) after adherent culture. Scale bar = 500 μm. (B) Immunostaining of three-dimensional (3D)-iPSC spheres for an undifferentiation state using the markers, TRA-1-60, SSEA4, and OCT4. High magnification images are shown as insets. Scale bar = 100 μm. (C) *In vitro* differentiation of 3D-iPSCs via embryoid bodies (EBs) expressing markers of the three germ layers. Immunostaining of markers of endoderm (AFP, green), mesoderm (α-SMA, green), and ectoderm (TUBB3, green) layers. Scale bar = 50 μm. (D) Teratoma assay for 3D-iPSCs derived from adipose-derived mesenchymal stem cells (AdSCs). Overall tumor appearance (left) and histological analysis (right). Hematoxylin and eosin staining revealed germ layer derivatives, such as neural tissues (Neu; ectoderm), cartilage (mesoderm), and gut epithelial tissues (Gut; endoderm). Scale bar = 200 μm. (E) Schematic summary of a TaqMan® Human Pluripotent Stem Cell Scorecard™ Panel assessment of 96 genes associated with self-renewal, endoderm, mesoderm, and ectoderm development for 2D- or 3D-iPSCs derived from AdSCs. A heat map and score box plot are shown in the upper panel. Expression plots represent fold change in expression of given genes compared to four samples. 2D-iPSCs and 3D-iPSCs represent undifferentiated states of each iPSC line, and 2D EBs or 3D EBs show self-differentiated states via EB formation from each iPSC line. A graph compares algorithm scores for the expression of given genes (lower panel). (F) Karyotype analysis of 3D-iPSCs at passage 9 using Q-banding. (G) Cell appearance 1 day after thawing (left) and after growth (right). Scale bar = 500 μm.

We conducted further analyses to assess the pluripotency of the newly established iPSCs under 3D conditions (3D-iPSCs). Some of the spheres were picked and seeded onto a Laminin-511 E8 fragment-coated dish in StemFit media [14]. The attached cells exhibited morphologies similar to those of human PSCs cultured in 2D conditions and stained for alkaline phosphatase (ALP; Fig. 2A). Immunostaining revealed expression of the undifferentiated state markers, TRA-1-60, SSEA4, and OCT4, in cells (Fig. 2B). Embryoid bodies (EB) were formed from 3D-iPSCs; they demonstrated self-differentiation into three germ layers as revealed by immunocytochemistry (Fig. 2C). They formed tumors following subcutaneous transplantation into immunodeficient mice, and the tumors contained tissues from all three germ layers (Fig. 2D). To compare the characteristics of iPSCs generated using the two different reprogramming methods, we performed a human pluripotent stem cell (hPSC) ScoreCard assay, which quantifies the ability of a human PSC line to differentiate into the three germ layers *in vitro* [16]. We used previously established iPSCs from the same parental AdSCs under 2D conditions (AdSC-derived 2D-iPSCs) as a control [9]. The ScoreCard assay indicated that both AdSC-derived 2D-iPSCs and 3D-iPSCs had similar characteristics; both iPSCs were in an undifferentiated state and possessed the ability to differentiate into three germ layers’ cells types via EB formation (Fig. 2E). Even after passaging, the spheroids increased in size, cleaved into sheets, self-dissociated into smaller pieces, and grew again (Fig. S1). The 3D-iPSCs maintained their growth for over 200 days and retained a stable 46,xx karyotype after prolonged culture at passage nine (Fig. 2F). Such 3D-iPSCs could be cryopreserved as cell aggregates using stem cell banker at −80 °C and were maintained after a normal thawing process (Fig. 2G).

The newly established 3D-iPSCs were in a pluripotent state similar to that of 2D-iPSCs generated using the conventional reprogramming method. Additionally, Q-banding karyotype of the 3D-iPSCs demonstrated a stable karyotype; the cells were cryopreserved while preserving their spheroid structures.

### 2.3 3D-iPSCs differentiate into neural and cardiac cells following lineage specifications in 3D culture and under enzymatic dissociation-free conditions

**Fig. 3:**
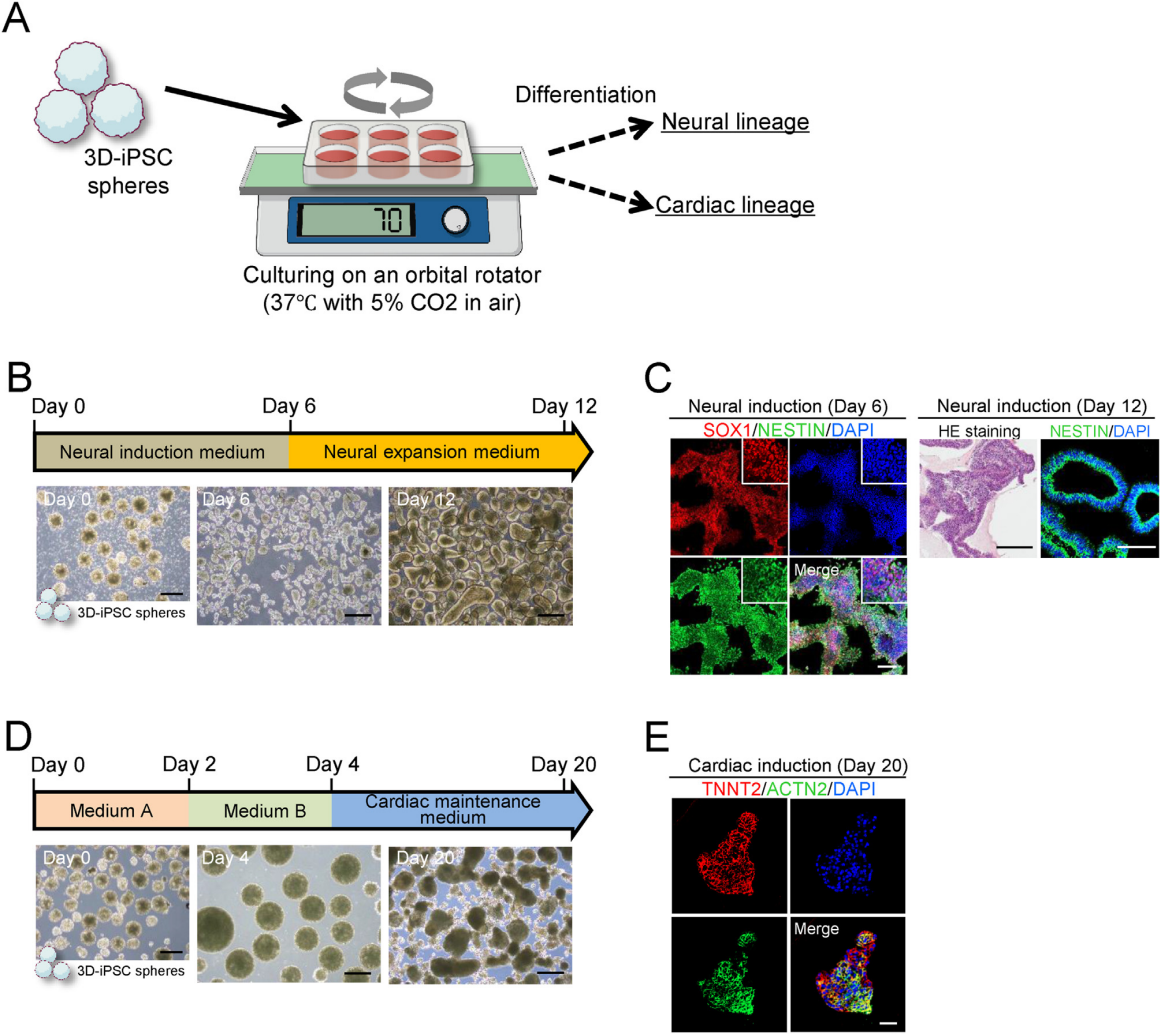
Lineage specification from 3D-iPSCs without enzymatic dissociation steps (A) Schema illustrating the lineage specification procedure from three-dimensional (3D)-induced pluripotent stem cells (iPSCs) under 3D conditions. 3D-iPSC spheres were just transferred to 6-well plates and cultured in differentiation medium on an orbital shaker at 37 °C, 5% CO_2_ in air. (B) Neural induction and analysis. Morphological changes and cell growth were observed by phase contrast microscopy during the induction process. Scale bar = 500 μm. (C) On day 6, spheres were attached and immunostained for the neural stem cell markers, SOX1 and NESTIN. Hematoxylin-eosin (HE) staining on day 12 revealed rosette-like structures within spheres. Immunostaining showed continued expression of neural stem cell markers. White scale bar = 100 μm, Black scale bar = 500 μm. (D) Cardiac induction and analysis. Morphological changes were observed by phase contrast microscopy during induction. Scale bar = 500 μm. (E) On day 20, differentiated cells were positively stained for the cardiac markers, alpha actin (ACTN2) and cardiac troponin T (TNNT2). Scale bar = 50 μm.

To facilitate the use of iPSCs in downstream applications, we employed specific protocols to induce neural and cardiac lineage differentiation. To establish a seamless workflow encompassing iPSC generation, cultivation, and differentiation, we implemented an orbital rotator system and induced differentiation under 3D conditions by transferring 3D-iPSC spheres directly into the differentiation medium (Fig. 3A). For neural lineage specification, we cultured 3D-iPSC spheres in a neural induction medium [6] (Fig. 3B). Immunostaining on day 6 following neural induction revealed expression of the neural stem cell markers, SOX1 and NESTIN, indicating successful neural lineage commitment. Upon cultivation in a neural stem cell expansion medium, we observed the neural formation of rosette-like structures through histological analysis. Immunostaining demonstrated cells continued to express NESTIN (Fig. 3C). For cardiac specification, we used a PSC cardiomyocyte differentiation kit [17] (Fig. 3D). At approximately day 20, beating cardiomyocyte-like cells were observed (data not shown). Immunostaining revealed the presence of the cardiomyocyte markers, actin (ACTN2) and cardiac troponin T (TNNT2), within these beating spheroids (Fig. 3E).

Three-dimensionally cultured- and enzymatic passage–free iPSC spheres can differentiate into specific lineages. These findings highlight the effectiveness of performing a series of processes, including somatic cell reprogramming, iPSC expansion, and differentiation, into specific cell types in a 3D-culture format without the need for enzymatic dissociation.

### 2.4 2D culture- and enzymatic passage-free reprogramming method for blood cells

**Fig. 4:**
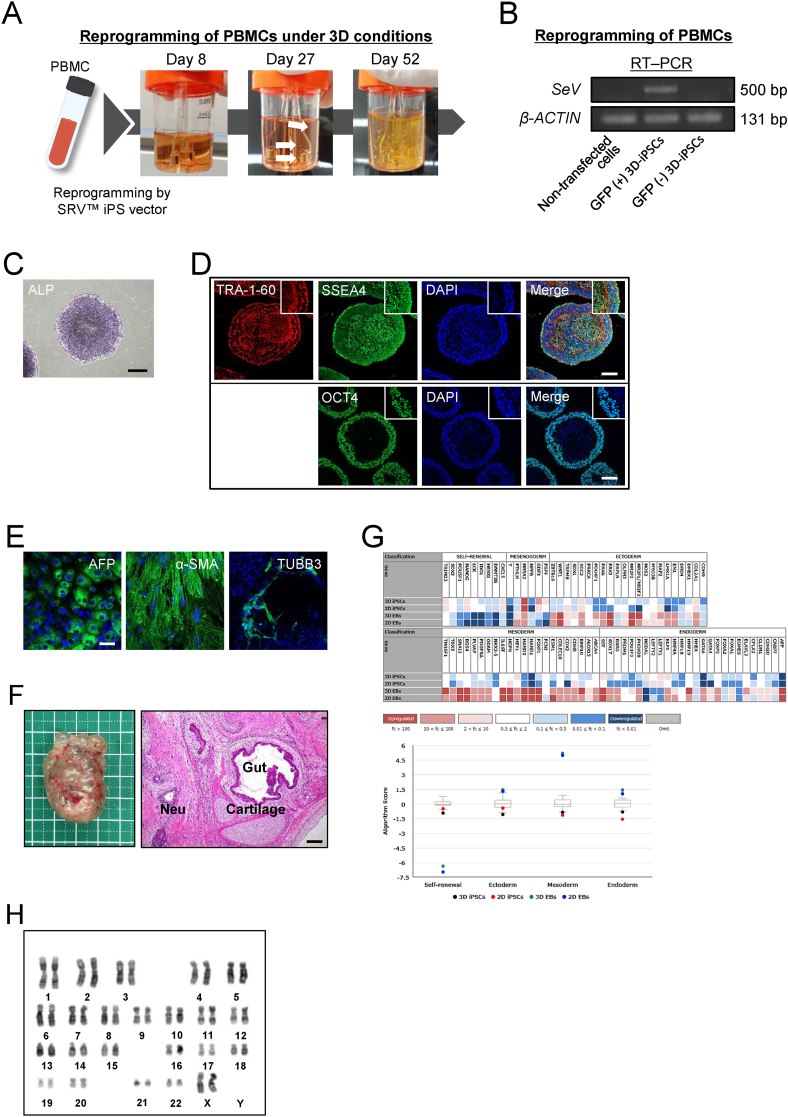
Generation and characterization of 3D-iPSCs from PBMCs (A) Workflow of the generation and expansion of peripheral blood mononuclear cell (PBMC)-derived three-dimensional (3D)-induced pluripotent stem cells (iPSCs) under 3D conditions. PBMCs were isolated from human blood and exposed to Sendai virus vector (SRV™ iPS vector). Cells were grown in small-scale (30 mL) spinner bioreactors at 37 °C in a 5% CO_2_ incubator. White arrows indicate primary spheres. (B) Quantitative reverse transcription polymerase chain reaction (RT–PCR) analysis of Sendai virus (*SeV*) and a housekeeping gene (*β-ACTIN*). Sendai virus vector was not detected in Green Fluorescent Protein (GFP)-negative 3D-iPSC cells by RT–PCR. (C) Alkaline phosphatase staining of 3D-iPSCs after adherent culture. Scale bar = 500 μm. (D) Immunostaining of markers for an undifferentiated state, TRA-1-60, SSEA4, and OCT4, in PBMC-derived 3D-iPSCs. Scale bar = 100 μm. (E) Immunostaining of differentiation markers, endoderm (AFP, green), mesoderm (α-SMA, green), and ectoderm (TUBB3, green) for adherent-cultured embryoid bodies (EBs) from PBMC-derived 3D-iPSCs. Scale bar = 50 μm. (F) Tumor formation assay after subcutaneous transplantation of PBMC-derived 3D-iPSCs. Tumor appearance (left). Histological analysis (right). Hematoxylin and eosin staining revealed intestinal tissue (Gut), cartilage tissue (Cartilage), and neural rosettes (Neu). Scale bar = 200 μm. (G) Schematic summary of a TaqMan® Human Pluripotent Stem Cell Scorecard™ Panel assessment of 96 genes associated with self-renewal, endoderm, mesoderm, and ectoderm development for 2D- or 3D-iPSCs derived from PBMCs. iPSCs generated from the same PBMCs using the same Sendai virus vector under 2D conditions (2D-iPSCs) were used as a control. A heat map and score box plot are shown (upper panel). Expression plots represent fold change in expression of given genes compared to four samples. 2D-iPSCs and 3D-iPSCs represent undifferentiated states of each iPSC line, and 2D EBs or 3D EBs showed self-differentiated states via EB formation from 2D- or 3D-iPSCs, respectively. A graph compares algorithm scores for the expression of given genes (lower panel). (H) Karyotype analysis of PBMC-derived 3D-iPSCs at passage six using Q-banding.

We determined the applicability of the newly-developed method for other somatic cell types. We aimed to generate iPSCs from peripheral blood mononuclear cells (PBMCs). To achieve this, we isolated PBMCs from human blood and performed PBMC reprogramming with a SRV™ iPSC Vector in suspension conditions for 2 h at 37 °C. The transfected cells were cultured in 30-mL spinner flasks using a stirred bioreactor system in StemScale media (Fig. 4A). Around day 27, cell spheres began to form. Similar to 3D-iPSC derivation from AdSCs, such blood-derived spheres increased in size and number without the need for single-cell dissociation. GFP-negative spheres did not contain SeV by reverse transcription polymerase chain reaction (RT–PCR; Fig. 4B). The cells were positive for ALP (Fig. 4C) and undifferentiated (Fig. 4D). These cells differentiated into all three germ layers through random differentiation via EB formation (Fig. 4E) and could form teratomas *in vivo* (Fig. 4F). Induced pluripotent stem cells were established using the same donor cells and viral vectors under 2D conditions (Fig. S2A–D). Using the TaqMan hPSC Scorecard assay, we compared PBMC-derived iPSCs in terms of their undifferentiated state and differentiation ability. The Scorecard assay revealed both PBMC-derived iPSCs (2D/3D) had similar characteristics: they were undifferentiated and could differentiate into three germ layers (Fig. 4G). In addition, the PBMC-derived 3D-iPSCs maintained a normal karyotype after prolonged cultivation (Fig. 4H). It is more convenient and less distressing for patients that blood cells to be used rather than fibroblasts as the cell source for iPSC research. Thus, this newly established iPSC workflow can also be applied to human blood cells.

## 3 Discussion

We established a novel platform for iPSC research that eliminates the need for 2D culture and enzymatic dissociation, thereby providing a streamlined and simplified workflow. The conventional iPSC research workflow has limitations in terms of robustness and scalability, with complex and cumbersome procedures. Reprogramming cells and establishing iPSCs require specialized skills [3], and maintaining and expanding iPSCs involve enzymatic or mechanical dissociation, even under 3D conditions [[4], [7]]. This enzymatic dissociation-free strategy overcomes these challenges by reducing technical and manual variability.

We observed the growth kinetics of 3D-iPSCs; when the spheres increased in size, the central part became filled with dead cells and spheres cleaved and fragmented into smaller pieces, consistent with previous reports [15]. The small clusters continued to proliferate, leading to an increase in the number and size of 3D-iPSC spheres. Despite the potential cell stress induced by such growth kinetics, 3D-iPSCs exhibited a normal karyotype, which is advantageous because karyotypic abnormalities can occur under stress conditions and repeated single-cell dissociation [[1], [2]].

Our 3D culture and enzymatic dissociation–free iPSC workflow eliminates the need for complicated procedures requiring specialized skills. This approach allows for the future automation of iPSC workflows, where fully automated machines can handle reprogramming, maintenance, cryopreservation, and differentiation under 3D conditions. This study outlines the initial steps toward the development of a fully-automated system for iPSC research.

We successfully reprogrammed human somatic cells under 3D conditions, demonstrating the feasibility of maintaining, expanding, cryopreserving, and differentiating 3D-iPSCs into neural and cardiac lineages. By eliminating the enzymatic dissociation step, our new iPSC generation and cultivation system may facilitate iPSC research in rare disease studies, regenerative medicine, and other applications.

## 4 Experimental procedure

### 4.1 Ethics statements

Human peripheral PBMCs were collected after obtaining written informed consent. All experiments were approved by the Institutional Review Board of the National Center for Child Health and Development (NCCHD) of Japan (permit nos. 385 and 396). All experiments involving human cells were performed in accordance with the tenets of the Declaration of Helsinki (revised 2013).

The animal protocol was approved by the Institutional Animal Care and Use Committee of the NCCHD (permit no. A2003-002). All animal experiments were based on the three Rs (refined, reduced, and replaced), with animal discomfort minimized and the number of animals used reduced.

### 4.2 Reprogramming somatic cells using Sendai virus vector

We used a commercially available Sendai virus vector, SRV™ iPSC Vector (TOKIWA-Bio Inc., Ibaraki, Japan) and SRV iPS-2 Vector (carrying OCT4, KLF4, SOX2, and C-MYC) for human AdSCs reprogramming, and an SRV iPS-4 vector (carrying OCT4, KLF4, SOX2, C-MYC, NANOG, and LIN28) for PBMC reprogramming according to the manufacturer's instructions. Both SRV vectors encoded the enhanced GFP reporter gene, along with reprogramming factors. Adipose-derived stem cells were from Lonza Bioscience (PT5006; Walkersville, MD, USA). Adipose-derived stem cells were cultured in ADSC basal medium (Lonza Bioscience) in a humidified atmosphere at 37 °C with 5% CO_2_ in air and then collected using TrypLE Select enzyme (Thermo Fisher Scientific, Waltham, MA, USA) for reprogramming procedures. Peripheral blood mononuclear cells were separated using a Leucosep™ System (Greiner Bio-One, Kremsmünster, Austria). Each blood sample was poured into a Leucosep tube and centrifuged for 15 min at 1000 × *g*. After discarding the plasma layer fraction, we harvested the enriched cell fraction into another centrifugation tube and washed it with Dulbecco's phosphate-buffered saline (DPBS, Thermo Fisher Scientific) two times.

Adipose-derived stem cells or PBMCs were suspended in a medium containing SRV™ iPSC vector at a multiplicity of transfection of 1 or 3. After a 2-h incubation at 37 °C, the cells were washed to remove Sendai virus. Subsequently, infected cells were suspended in 30-mL spinner flasks (ABLE Biott, Tokyo, Japan) with StemScale™ PSC Suspension Medium (Thermo Fisher Scientific) and cultured in a single-use bioreactor (ABLE Biott). To facilitate the reprogramming process, we added two small molecules: 5 μM DAPT (NOTCH1 inhibitor; R&D Systems, Minneapolis, MN, USA) and 3 μM iDOT1L (Abcam, Cambridge, UK).

### 4.3 Suspension cultures of human iPSCs

Human iPSCs were cultured in a 30-mL single-use bioreactor (ABLE Biott) at 37 °C with 5% CO_2_ in air and agitated at 55 rpm. The cells were suspended in 30 mL of StemScale™ PSC Suspension Medium (Thermo Fisher Scientific). Half of the medium was replaced every alternate day. Upon growth, some of the primary spheres were transferred to the next bioreactor as passages.

## 5 Characterization of 3D-iPSCs

### 5.1 Alkaline phosphatase staining

Three dimensional–induced pluripotent stem cell spheres were placed onto an adhesion culture dish and fixed in 4% paraformaldehyde for 20 min at 4 °C. Fixed cells were stained with BCIP/NBT (5-bromo-4-chloro-3-indolyl-phosphate/nitro blue tetrazolium) solution (Nacalai Tesque, Kyoto, Japan) according to the manufacturer's instructions. Images were acquired using a BZ-X700 microscope (Keyence, Osaka, Japan).

### 5.2 EB formation for *in vitro* differentiation assay

Human iPSC colonies were washed with DPBS, and cells were collected using TrypLE Select. Dissociated cells were used to seed in Costar® Ultra Low Cluster 96 Well Round Bottom Plate (Corning, Inc., New York, NY, USA) at a density of 1.0 × 10^4^ cells/well in DMEM/F12-based medium with 20% fetal bovine serum (FBS), 2 mM L-glutamine, 1 mM sodium pyruvate, 0.1 mM nonessential amino acids, 100 U/mL penicillin, and 100 μg/mL streptomycin (all reagents from Thermo Fisher Scientific). The resulting EB cultures were maintained in 96-well plates for 7 days and then replated onto glass-bottom dishes coated with 0.1% gelatin (Sigma-Aldrich, Darmstadt, Germany) for a further 14 days.

### 5.3 Quantitative RT–PCR analysis

Total RNA was extracted from the cell pellet using an RNeasy Mini kit (Qiagen, Hilden, Germany), and DNA was removed using DNase (Thermo Fisher Scientific). First-strand complementary DNA (cDNA) was synthesized using SuperScript IV VILO (Thermo Fisher Scientific). Olymerase chain reaction or quantitative PCR was performed using TaKaRa Ex Taq DNA Polymerase (Takara, Shiga, Japan) or TaqMan™ Gene Expression Master Mix (Thermo Fisher Scientific) in a ProFlex PCR System or QuantStadio 7 Real-Time PCR System thermal cyclers (Thermo Fisher Scientific). To detect Sendai virus (SeV) by RT–PCR, specific primer sets were used: *SeV* (500 bp) forward; 5′-ATATGGAGTACGAGAGGACC-3′, reverse; 5′-CCTCAGGTTGGAGAGAGTCA-3′, *β-ACTIN* (131bp) forward; 5′-TCCCTGGAGAAGAGCTACG-3′, reverse; 5′-GTAGTTTCGTGGATGCCACA-3’.

### 5.4 Human pluripotent stem cell Scorecard assay

The cDNA was prepared using SuperScript IV VILO (Thermo Fisher Scientific). TaqMan® Human Pluripotent Stem Cell (hPSC) Scorecard™ assays were performed according to the manufacturer's instructions (Thermo Fisher Scientific). The hPSC Scorecard assay was used to investigate iPSC pluripotency by assessing expression levels of genes that play a key role in self-renewal, endoderm, mesoderm, and ectoderm development. Gene expression data was analyzed using hPSC Scorecard™ Analysis Software (Thermo Fisher Scientific).

### 5.5 Teratoma formation for *in vivo* differentiation assay

Approximately 1–5 × 10^7^ cells were subcutaneously transplanted into nude mice (BALB/cAJcl-nu/nu; CLEA Japan, Tokyo, Japan). Tumor masses were collected after 2–3 months and fixed with 4% paraformaldehyde, paraffin embedded, sectioned into 5-μm sections, and stained with hematoxylin and eosin. Tumor portions were subjected to histological analysis; the three germ layers were identified based on representative histological features.

### 5.6 Immunofluorescence staining

The 3D-iPSC spheres were fixed with formalin and embedded in paraffin. Sectioned samples were incubated with primary antibodies at 4 °C overnight. After washing with DPBS, samples were incubated for 30 min at 25 °C with secondary antibodies conjugated to Alexa 488 or 546 (Thermo Fisher Scientific). After washing with DPBS, mounting medium containing DAPI was used. Primary and secondary antibodies used are listed in Table S. Images were acquired with a confocal laser microscopy (LSM900; Carl Zeiss, Oberkochen, Germany).

### 5.7 Neural and cardiac lineage induction from 3D-iPSCs

To induce differentiation into neural or cardiac lineages, cells were cultured in an orbital shaker (MaxQ 2000 CO_2_ Plus, Thermo Fisher Scientific) at 70 rpm. For neural induction, 3D-iPSC spheres were transferred to 6-well culture dishes and cultured in PSC neural induction medium (A1647801; Thermo Fisher Scientific). On day 6, neural induction medium was replaced with neural expansion medium (A1647801; Thermo Fisher Scientific) and cells were cultured for an additional six days. For cardiac induction, 3D-iPSC spheres were transferred to 6-well culture dishes and cultured using a PSC cardiomyocyte differentiation kit (A2921201; Thermo Fisher Scientific) according to the manufacturer's instructions. The medium was changed every other day. On day 20, spheres were embedded in paraffin, sectioned, and immunolabeled for cardiac markers.

### 5.8 Karyotypic analysis

Chromosomal Q-band analyses of 3D-iPSCs were performed by Chromosome Science Labo. Ltd. (Sapporo, Hokkaido, Japan). At least 20 metaphase spreads were examined for each cell line.

## Author contributions

Conceptualization, H.A.; Methodology, M.T., T.K., and H.A.; Investigation, M.T.; Writing-Original Draft, M.T.; Writing-Review and Editing, M.C.V., A.U., and H.A.; Visualization, M.T. and T.K.; Supervision, A.U. and H.A.

## Declaration of competing interest

The authors have no conflicts of interest to report.
