# Supplementary material for: A passage-free, simplified, and scalable novel method for iPSC generation in three-dimensional culture
Source: Regen Ther. 2024 Mar 10;27:39–47. doi: 10.1016/j.reth.2024.02.005 (PMC10940796; doi:10.1016/j.reth.2024.02.005)
Supplement: Multimedia component 1 [file mmc1.pptx]

## Slide 1
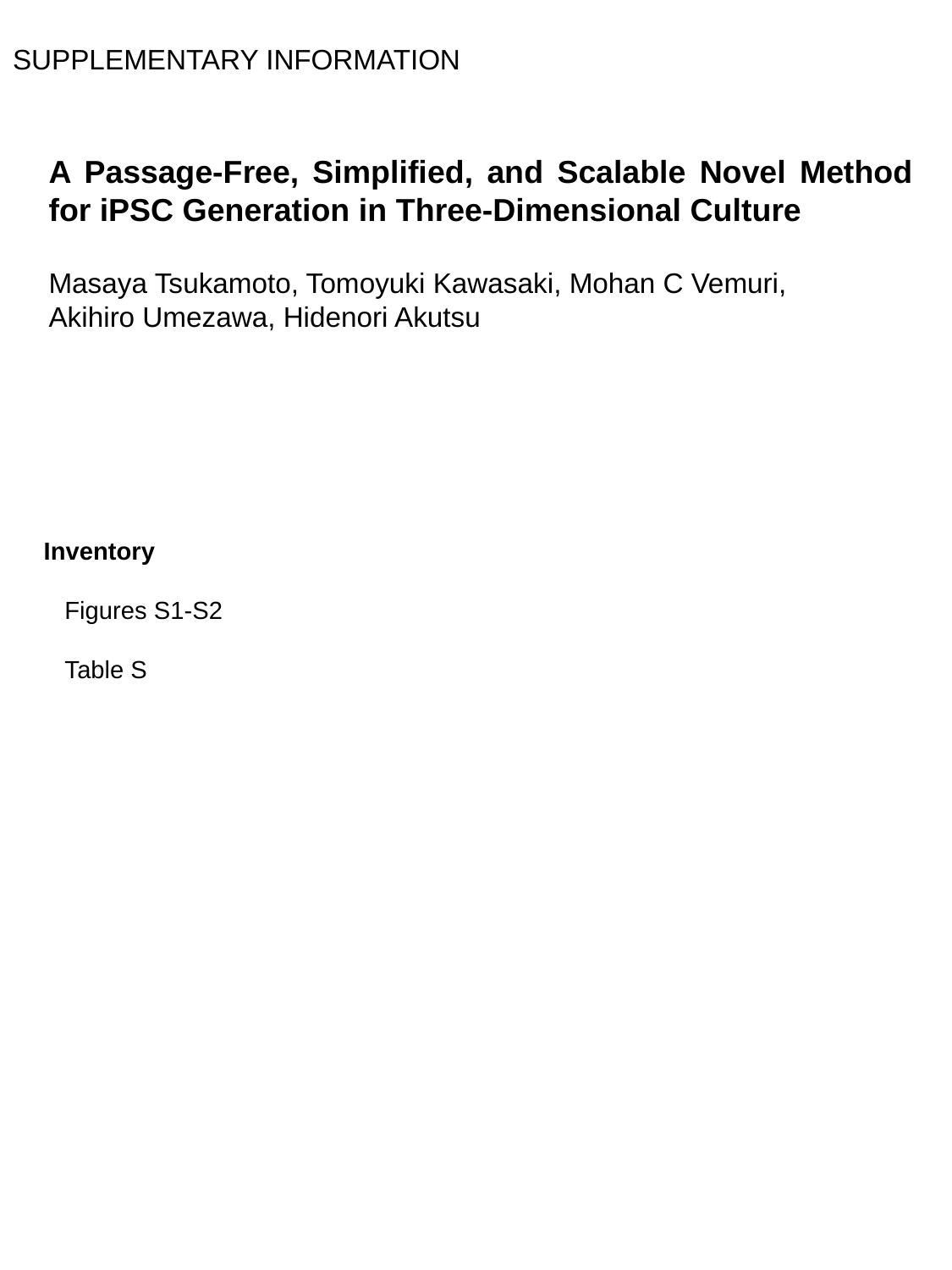

# SUPPLEMENTARY INFORMATION
A Passage-Free, Simplified, and Scalable Novel Method for iPSC Generation in Three-Dimensional Culture
Masaya Tsukamoto, Tomoyuki Kawasaki, Mohan C Vemuri,
Akihiro Umezawa, Hidenori Akutsu
Inventory
 Figures S1-S2
 Table S

## Slide 2
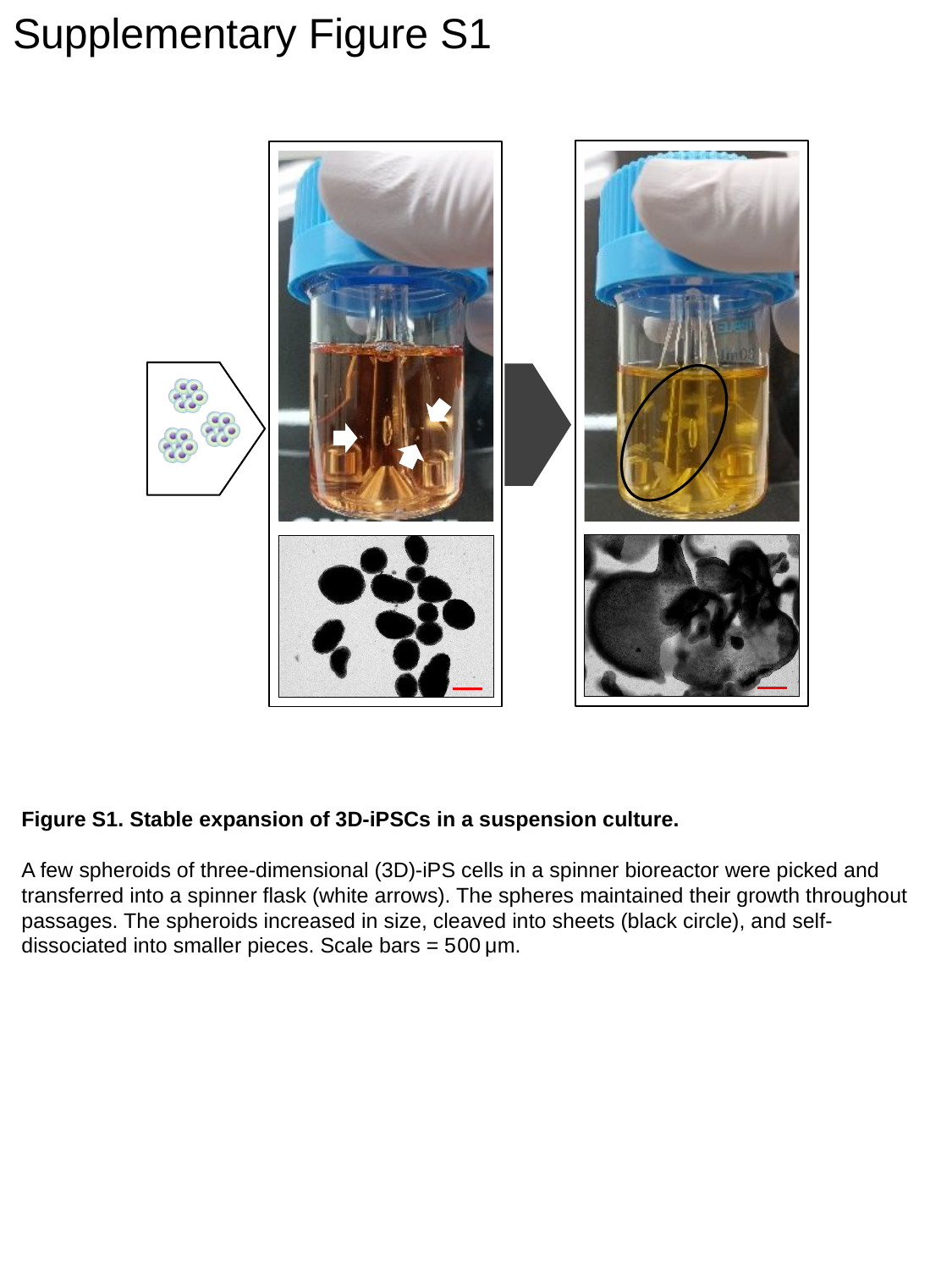

# Supplementary Figure S1
Figure S1. Stable expansion of 3D-iPSCs in a suspension culture.
A few spheroids of three-dimensional (3D)-iPS cells in a spinner bioreactor were picked and transferred into a spinner flask (white arrows). The spheres maintained their growth throughout passages. The spheroids increased in size, cleaved into sheets (black circle), and self-dissociated into smaller pieces. Scale bars = 500 μm.

## Slide 3
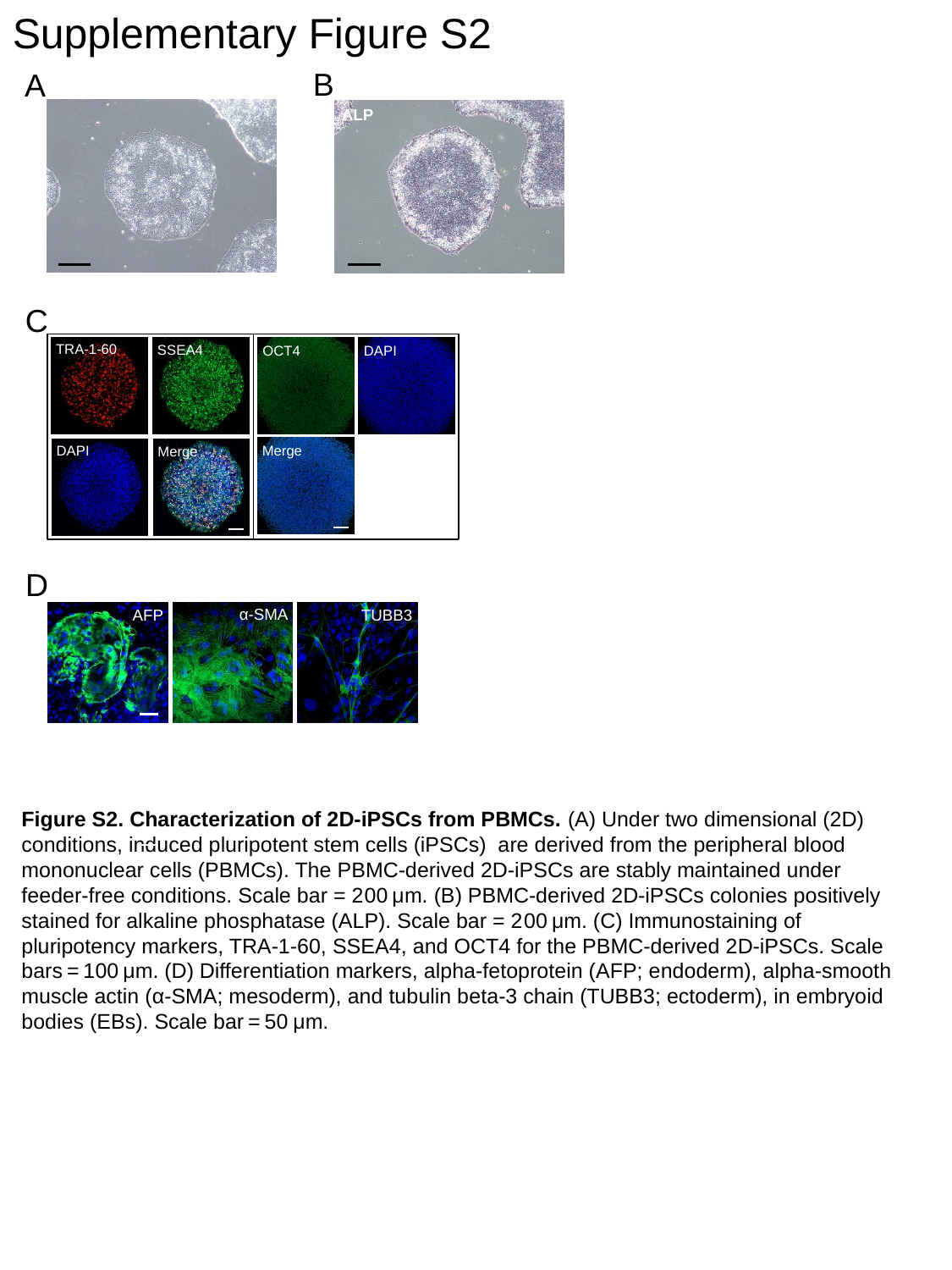

Supplementary Figure S2
B
A
ALP
C
TRA-1-60
OCT4
SSEA4
DAPI
OCT4
Merge
DAPI
Merge
D
α-SMA
TUBB3
AFP
Figure S2. Characterization of 2D-iPSCs from PBMCs. (A) Under two dimensional (2D) conditions, induced pluripotent stem cells (iPSCs) are derived from the peripheral blood mononuclear cells (PBMCs). The PBMC-derived 2D-iPSCs are stably maintained under feeder-free conditions. Scale bar = 200 μm. (B) PBMC-derived 2D-iPSCs colonies positively stained for alkaline phosphatase (ALP). Scale bar = 200 μm. (C) Immunostaining of pluripotency markers, TRA-1-60, SSEA4, and OCT4 for the PBMC-derived 2D-iPSCs. Scale bars = 100 μm. (D) Differentiation markers, alpha-fetoprotein (AFP; endoderm), alpha-smooth muscle actin (α-SMA; mesoderm), and tubulin beta-3 chain (TUBB3; ectoderm), in embryoid bodies (EBs). Scale bar = 50 μm.

## Slide 4
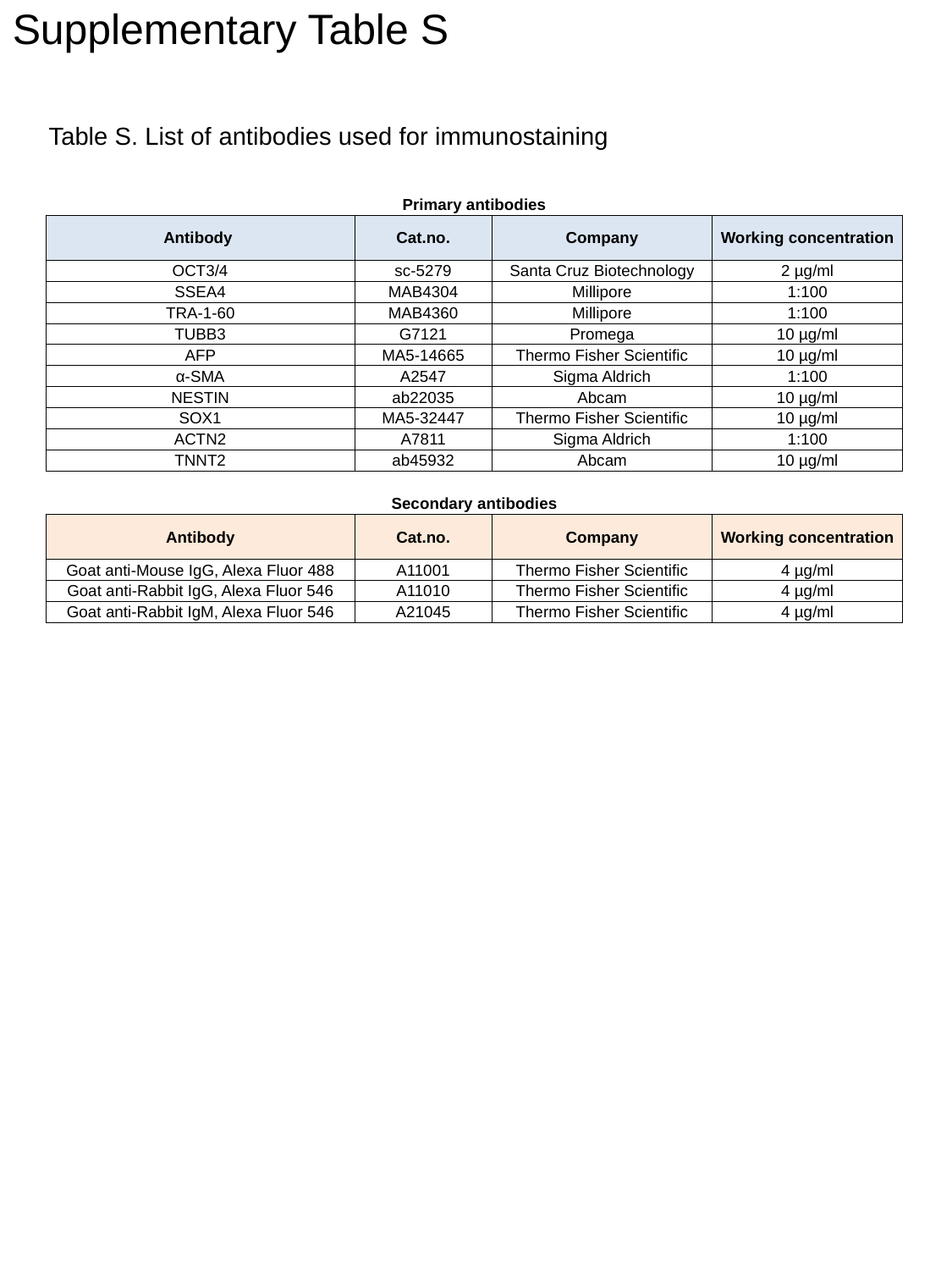

Supplementary Table S
Table S. List of antibodies used for immunostaining
| Primary antibodies | | | |
| --- | --- | --- | --- |
| Antibody | Cat.no. | Company | Working concentration |
| OCT3/4 | sc-5279 | Santa Cruz Biotechnology | 2 µg/ml |
| SSEA4 | MAB4304 | Millipore | 1:100 |
| TRA-1-60 | MAB4360 | Millipore | 1:100 |
| TUBB3 | G7121 | Promega | 10 µg/ml |
| AFP | MA5-14665 | Thermo Fisher Scientific | 10 µg/ml |
| α-SMA | A2547 | Sigma Aldrich | 1:100 |
| NESTIN | ab22035 | Abcam | 10 µg/ml |
| SOX1 | MA5-32447 | Thermo Fisher Scientific | 10 µg/ml |
| ACTN2 | A7811 | Sigma Aldrich | 1:100 |
| TNNT2 | ab45932 | Abcam | 10 µg/ml |
| | | | |
| Secondary antibodies | | | |
| Antibody | Cat.no. | Company | Working concentration |
| Goat anti-Mouse IgG, Alexa Fluor 488 | A11001 | Thermo Fisher Scientific | 4 µg/ml |
| Goat anti-Rabbit IgG, Alexa Fluor 546 | A11010 | Thermo Fisher Scientific | 4 µg/ml |
| Goat anti-Rabbit IgM, Alexa Fluor 546 | A21045 | Thermo Fisher Scientific | 4 µg/ml |
